# Identification and Characterization of a Novel *Plasmodium falciparum* Adhesin Involved in Erythrocyte Invasion

**DOI:** 10.1371/journal.pone.0074790

**Published:** 2013-09-13

**Authors:** Nidhi Hans, Shailja Singh, Alok K. Pandey, K. Sony Reddy, Deepak Gaur, Virander S. Chauhan

**Affiliations:** Malaria Research Group, International Centre for Genetic Engineering and Biotechnology (ICGEB), New Delhi, India; Université Pierre et Marie Curie, France

## Abstract

Malaria remains a major health problem worldwide. All clinical symptoms of malaria are attributed to the asexual blood stages of the parasite life cycle. Proteins resident in apical organelles and present on the surface of *P. falciparum* merozoites are considered promising candidates for the development of blood stage malaria vaccines. In the present study, we have identified and characterized a microneme associated antigen, PfMA [PlasmoDB Gene ID: PF3D7_0316000, PFC0700c]. The gene was selected by applying a set of screening criteria such as transcriptional upregulation at late schizogony, inter-species conservation and the presence of signal sequence or transmembrane domains. The gene sequence of PfMA was found to be conserved amongst various *Plasmodium* species. We experimentally demonstrated that the transcript for PfMA was expressed only in the late blood stages of parasite consistent with a putative role in erythrocyte invasion. PfMA was localized by immunofluorescence and immuno-electron microscopy to be in the micronemes, an apical organelle of merozoites. The functional role of the PfMA protein in erythrocyte invasion was identified as a parasite adhesin involved in direct attachment with the target erythrocyte. PfMA was demonstrated to bind erythrocytes in a sialic acid independent, chymotrypsin and trypsin resistant manner and its antibodies inhibited *P. falciparum* erythrocyte invasion. Invasion of erythrocytes is a complex multistep process that involves a number of redundant ligand-receptor interactions many of which still remain unknown and even uncharacterized. Our work has identified and characterized a novel *P. falciparum* adhesin involved in erythrocyte invasion.

## Introduction

Malaria still remains a major infectious disease that plagues the world despite extensive efforts spanning more than a century to control this disease. Every year about 300–500 million malaria cases are detected that lead to about 1 million deaths worldwide [1]. Most of the clinical symptoms of *P. falciparum* malaria are attributed to asexual propagation of the parasite within human erythrocytes. The blood stage life cycle involves merozoite invasion, growth, multiplication within the infected erythrocyte (schizogony), followed by egress of the daughter merozoites that go onto invade new uninfected erythrocytes. Thus parasite entry into the host erythrocyte is the most critical step of its life cycle with respect to malaria pathogenesis. It involves primary contact via proteins coated on its surface, followed by release of proteins resident in apical secretory organelles to form a tight junction [2-4]. The tight junction powered by the parasite actin-myosin motor moves as a circumferential ring along the parasite-erythrocyte interface facilitating merozoite entry into the host cell. During the invasion process, the parasite creates a parasitophorous vacuole within which it resides in the host erythrocyte [2,3].

*P. falciparum* erythrocyte invasion involves a number of redundant ligand-receptor interactions that allow the parasite to invade through multiple alternate pathways [2-4]. The *P. falciparum* genome encodes 5300 genes of which an estimated 2700 are expressed during the blood stages [5,6]. The entire set of parasite ligands involved in erythrocyte invasion still remains unknown. Understanding the complex process of *P. falciparum* merozoite invasion requires the identification and characterization of novel parasite ligands and their interactions with receptors on erythrocytes. The availability of the *P. falciparum* transcriptome data has provided opportunities for the identification of novel antigens, which are involved in merozoite invasion [5-7].

Transcriptome analysis of the complete asexual intraerythrocytic developmental cycle (IDC) of *P. falciparum* identified 262 ORFs that showed an expression induction during late schizont stages similar to leading malaria vaccine candidates and other well characterized merozoite surface/apical proteins that are known to play a role in erythrocyte invasion [6]. Of the 262 ORFs, 189 were of unknown function, representing a list of novel putative antigens. Recent reports also enlist an invadome sub-network wherein 418 genes have been hypothesized to be involved in *P. falciparum* merozoite invasion [7]. However their localization and functional role in merozoite invasion process remains to be elucidated.

In the current study, we have identified a novel *P. falciparum* merozoite protein, PF3D7_0316000 (PFC0700c) and have characterized its role in erythrocyte invasion. The protein was demonstrated to be expressed at the schizont stage of the asexual life cycle, localized in the micronemes and thus named as PfMA (*P. falciparum* Microneme Associated Antigen). PfMA was identified as a novel parasite adhesin that exhibited specific erythrocyte binding activity and whose antibodies blocked erythrocyte invasion. This study has validated the functional role of a novel parasite protein (PfMA) in erythrocyte invasion.

## Results

### Identification and sequence analysis of PF3D7_0316000- a *P. falciparum* microneme associated antigen (PfMA)

**Figure 1:**
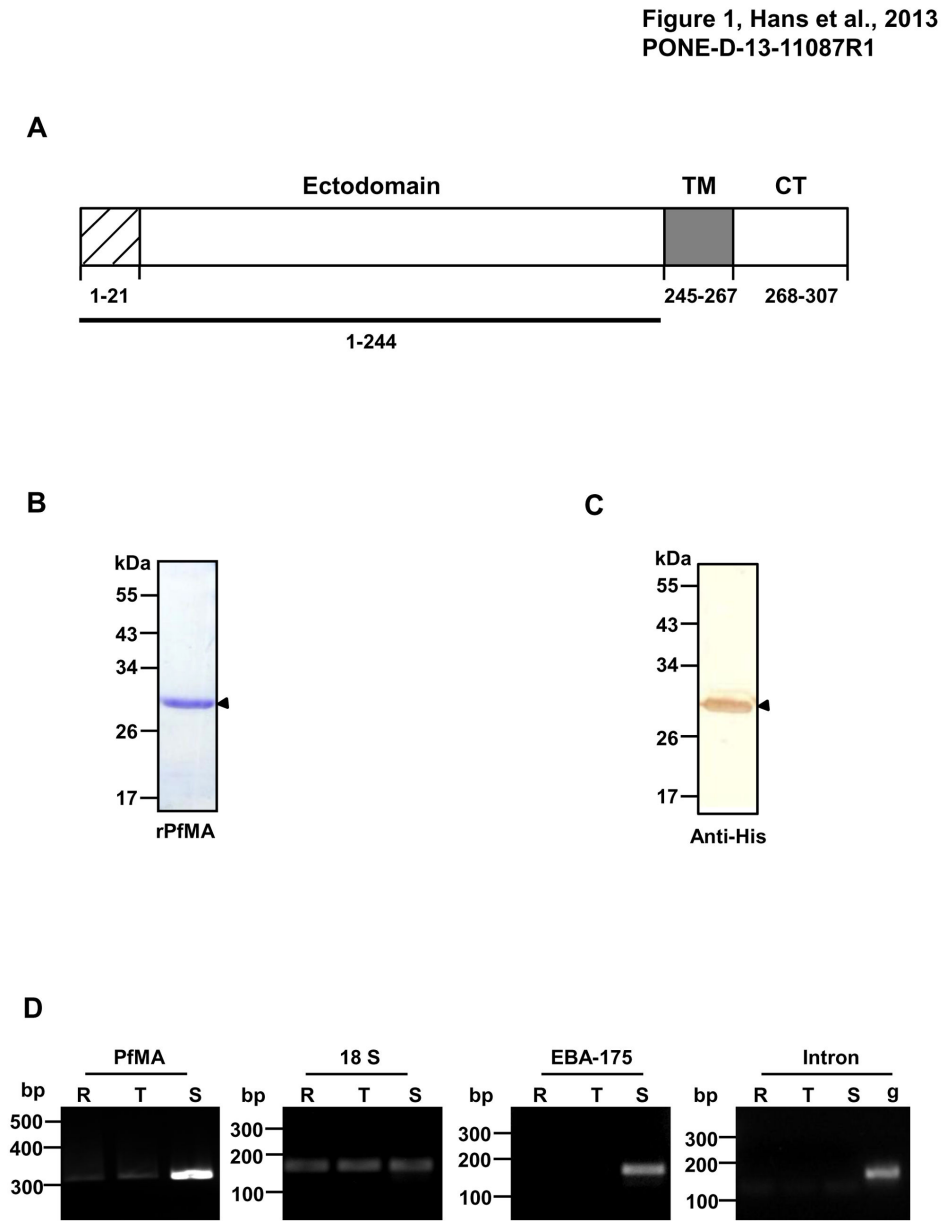
Expression of recombinant PfMA and transcriptional analysis of PfMA in different blood stages of *P. falciparum*. (**A**) Schematic representation of the primary structure of the 307 amino acid PfMA protein (PlasmoDB ID PF3D7_0316000). PfMA comprises of an N-terminal hydrophobic stretch (residues 1 to 21), a transmembrane TM domain (residues 245-267), and a cytoplasmic tail (CT) at the carboxyl terminal (residues 268-307). The ectodomain (1-244 residues) was expressed in a recombinant form (shown as a bar) and antibodies were raised to this region. (**B**) SDS-PAGE showing purified recombinant PfMA (rPfMA) at the expected size of 30kDa (shown with arrow). (**C**) Detection of rPfMA by anti-polyHistidine tag antibody (Sigma) by immunoblotting (shown with arrow) (**D**) RT-PCR analysis of PfMA in different blood stages of *P. falciparum* cDNA prepared from different blood stages i.e. ring (R), trophozoite (T) and schizont (S) were used to amplify PfMA transcripts using gene specific primers. The level of PfMA transcription increased from the ring to schizont stages. As a control for equal amounts of RNA from the three stages, RT-PCR was performed with the 18S rRNA primers. RT-PCR of EBA-175 was analyzed as a control for stage specificity. Intronic primers showed amplification only in genomic DNA (g) and not in cDNA from all the stages.

In our efforts to identify novel merozoite proteins that might be involved in erythrocyte invasion, we screened three transcription databases to identify genes whose expression profile matched that of well characterized genes known to be involved in the invasion process [5-7]. These genes were further sorted, on the basis of their conservation among different *Plasmodium* species and the presence of either a signal peptide or transmembrane domain in their encoded proteins. On this basis, PF3D7_0316000 was selected for further validation of its role in erythrocyte invasion. PF3D7_0316000 is a 307 amino acid protein (~ 37.1 kDa) that contains an N-terminal stretch of hydrophobic residues, a C-terminal single transmembrane domain and a short cytoplasmic tail. A schematic representation of the PfMA protein is represented in Figure 1A. PfMA homologues were identified in *P. vivax* (PVX_095435), *P. berghei* (PBANKA_041380), *P. chabaudi* (PCHAS_041470), *P* *. yoelii* (PY03459) and *P* *. cynomolgi* (PCYB_083370). An alignment of the predicted protein sequences of these putative MA genes from different *Plasmodium* species (Figure S1) showed a 22.9% identity in terms of the number of identical residues amongst the six PfMA orthologues. A pair wise identity of 46.2% was analyzed by the Geneious R6 software (Biomatters) that represents the average identity of all possible pairs between the species.

### Expression of recombinant PfMA

The DNA sequence of the PfMA gene encoding a 244 amino acid region of the ectodomain of the parasite protein was cloned in the T7 promoter based plasmid pET-28a(+) (Novagen). The recombinant protein was expressed in *E. coli* BL21 (DE3) cells (Codon plus, Stratagene) with a 6×His-tag. The protein got expressed in inclusion bodies and was purified by metal affinity chromatography (Ni-NTA column) and followed by removal of the denaturant by dilution in an L-Arginine based Tris buffer pH 7.4. The protein was further dialyzed in 50 mM Tris pH 7.4 and 150 mM NaCl. The purified recombinant PfMA protein (rPfMA) migrated as a single band on SDS-PAGE (Figure 1B), which was also detected by immunoblotting using an anti-polyHistidine tag antibody (Sigma) (Figure 1C). Reverse phase-HPLC analysis of rPfMA on a C8 column showed a single symmetrical peak indicating that rPfMA had been purified to homogeneity (Figure S2A). rPfMA was subjected to trypsin digestion followed by MALDI/TOF/TOF. The mass spectrometric analysis revealed peptides corresponding to rPfMA that confirmed its identity (Figure S2B). The purified protein was formulated with complete Freund’s adjuvant or incomplete Freund’s adjuvant and used to immunize mice and rabbit. Terminal bleeds were collected on Day 70 and the antibody titres were analyzed by ELISA. Both mice and rabbit sera showed end point titres for the PfMA antibodies around 1:240000 (Figure S3A,B). The ELISA results represent an average of two independent experiments performed in triplicate. These antibodies detected rPfMA in immunoblotting, in which pre-immune antibodies failed to detect rPfMA confirming the specificity of anti-PfMA antibodies (Figure S3C).

### Expression analysis of PfMA during the different stages of the asexual erythrocytic life cycle

To confirm the expression of PfMA transcripts during the asexual blood stage of the parasite’s life cycle, cDNA were prepared from synchronized cultures (*P. falciparum* clone 3D7) at the ring, trophozoite and schizont stage parasites and analyzed by RT-PCR. Maximum expression of PfMA transcripts were observed at the schizont stage with a much lower expression detected in ring and trophozoite stages (Figure 1D). As a control for stage specificity, the transcript for EBA-175 was detected only in the schizont stage preparation as reported previously (Figure 1D) [8]. As a loading control, RT-PCR was performed with 18S rRNA primers that showed equal transcript expression at all three stages indicating that an equal amount of cDNA was used from the three different stages (Figure 1D). RT-PCR analysis with intron specific primers was performed as a control for checking any possible genomic DNA contamination in our cDNA preparations. The results clearly showed amplification of the 180 bp intron region only with genomic DNA and not with cDNA preparations (Figure 1D). Thus, we experimentally demonstrated that PfMA transcription followed a cyclic pattern during the blood stage life cycle with peak expression at the schizont stage. These findings are consistent with the expression profile observed with other *P. falciparum* genes known to be involved in erythrocyte invasion [5-7].

**Figure 2:**
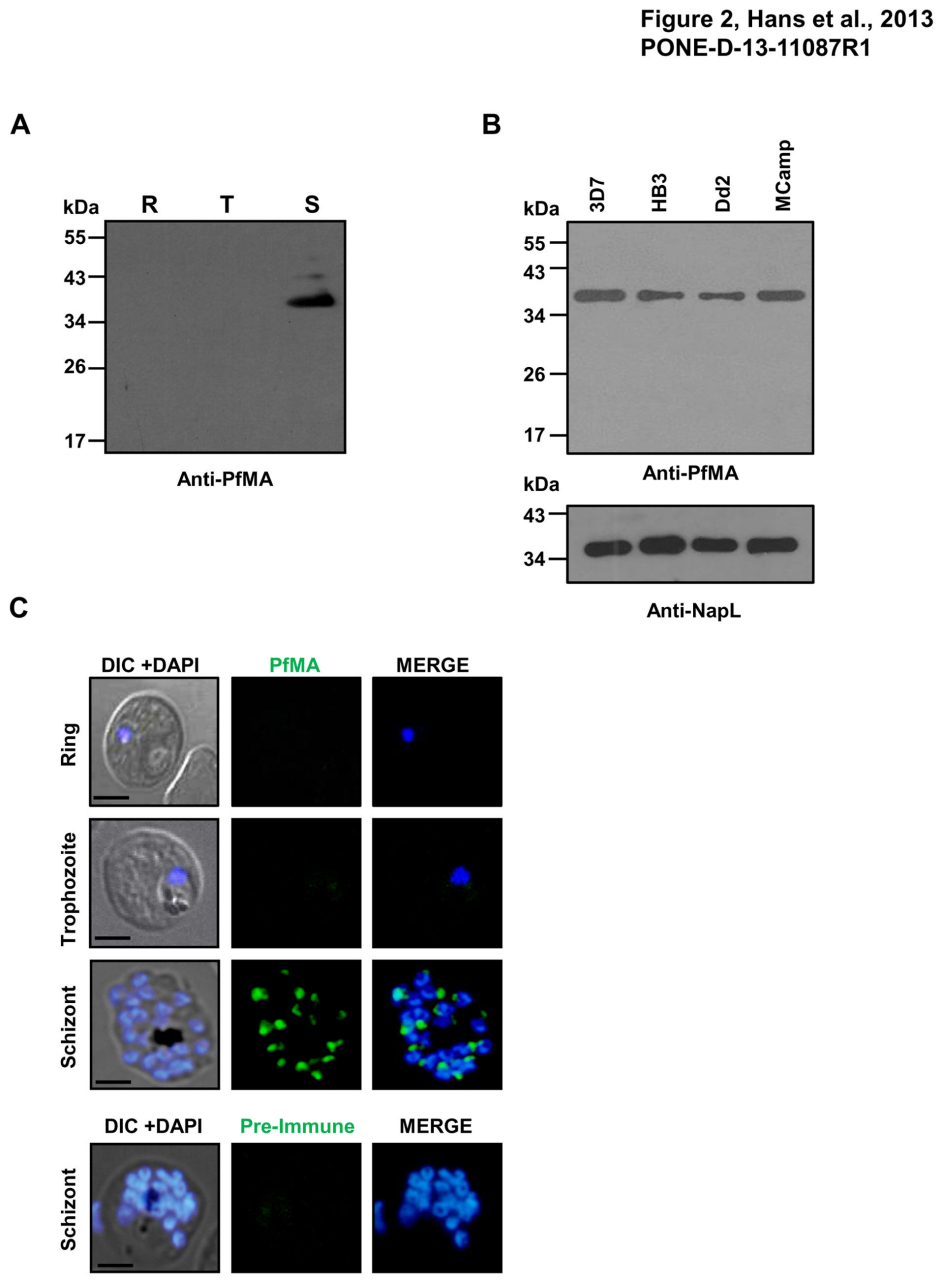
Protein expression of PfMA in different blood stages of *P. falciparum*. (**A**) Immunoblot analysis of equal amount of total protein from highly synchronized 3D7 parasites at ring (R), trophozoite (T) and schizont stage (S) with anti-PfMA antibodies showed a band in the schizont stage preparation at ~ 35 kDa representing full length fragment of PfMA. (**B**) Immunoblot analysis of PfMA expression amongst different *P. falciparum* strains showed a band of ~ 35 kDa similar to that of 3D7. The *P. falciparum* nuclear accessory protein NapL was probed as a loading control. (**C**) PfMA protein expression was analyzed in different stages of the asexual blood-stage life cycle by confocal immune-fluorescence microscopy using anti-PfMA mouse serum (green). Parasite nuclei were counterstained with DAPI (blue). No detectable PfMA staining was observed in ring and trophozoite stages. In schizonts, PfMA was detected as a punctate staining characteristic of many apical organelle resident proteins. No staining was detected with pre-immune sera. The scale bar indicates 2 µm.

To further confirm PfMA expression at the protein level, synchronised parasites (*P. falciparum* 3D7 clone) from the three stages [ring (R), trophozoite (T), schizont (S)] were harvested, treated with saponin and the total proteins were extracted using the detergent based RIPA lysis buffer. Equal amounts of the total protein lysates from the three parasite stages were subjected to immunoblotting using specific anti-PfMA antibodies. A ~35kDa band corresponding to the expected size of the native parasite protein was detected only in the schizont stage consistent with our RNA transcription data described above (Figure 2A). In order to ensure equal loading of the three lysates, we performed a BCA estimation of the protein content in each of the three lysate samples and loaded an equal amount (30 µg) on the gel (Figure S3D)

PfMA protein expression was also analyzed in three other *P. falciparum* clones, HB3, Dd2 and MCamp by immunoblotting using anti-PfMA antibodies. A ~35kDa band similar to that observed in 3D7 was also detected in the three strains confirming PfMA expression among these strains (Figure 2B). The nucleosome assembly protein, NapL [9] was probed as a control in the immunoblot experiment.

The expression of PfMA protein was also evaluated by immunofluorescence confocal microscopy. PfMA expression was detected in the schizont stage and neither in the ring or trophozoite stage parasites (Figure 2C). No fluorescence staining was observed in the schizont stage with pre-immune sera (Figure 2C). In schizonts, PfMA showed punctate staining towards the apex of individual merozoites consistent with the staining observed with invasion proteins known to reside in the apical organelles [10-14]. These results confirm that PfMA is expressed during the schizont stage of the erythrocytic life cycle of the parasite.

### Sub-cellular localization of PfMA in *P. falciparum*

**Figure 3:**
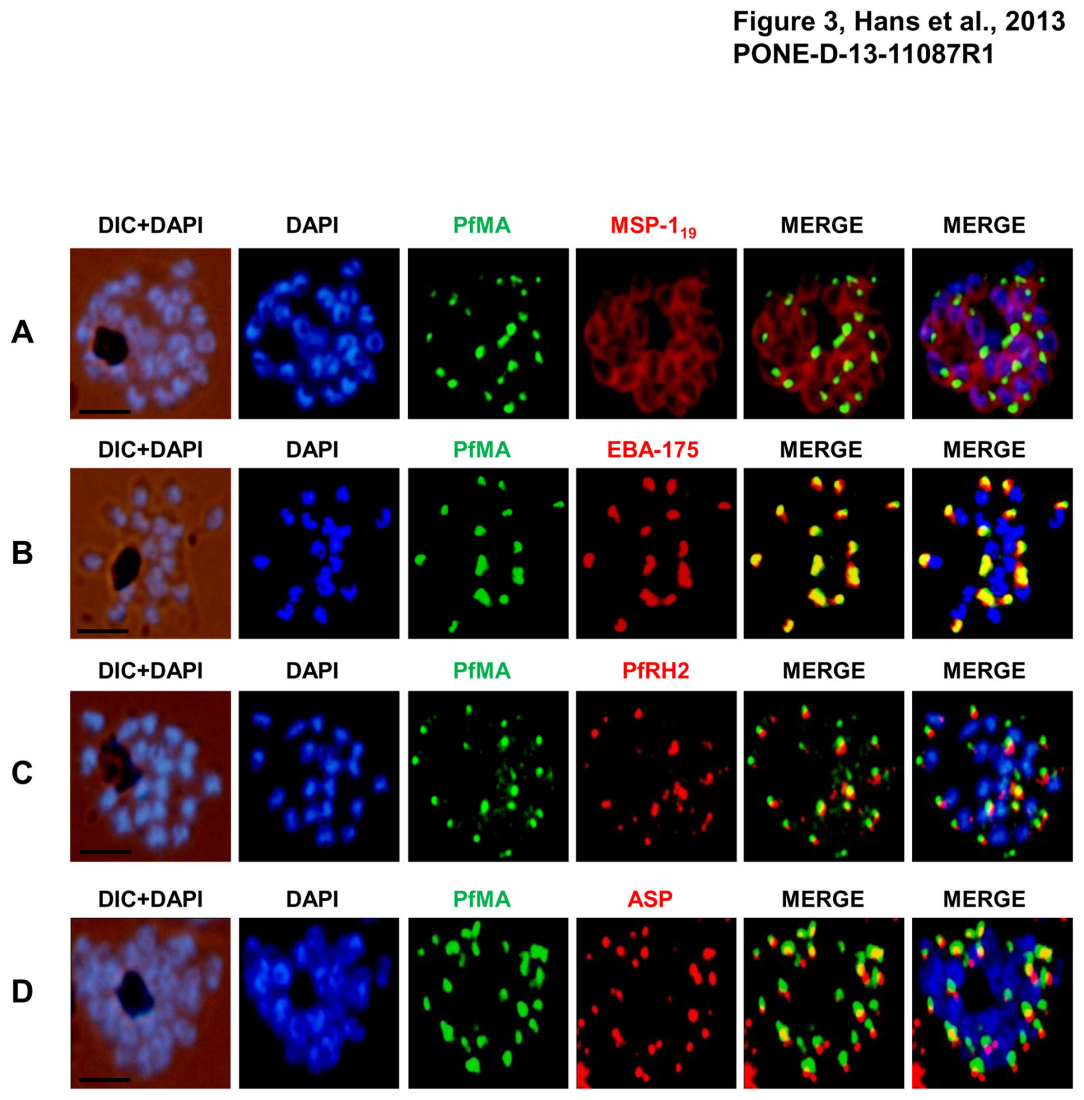
PfMA localization in schizont stages analyzed by fluorescence microscopy. Sub-cellular localization of PfMA was studied by co-immunostaining with surface protein (**A**), microneme (**B**), rhoptry (**C**, **D**) resident proteins. *P. falciparum* schizonts were co-immunostained with mouse anti-PfMA (green) and rabbit antibodies against one of the 4 marker proteins (EBA175/PfRH2, ASP, MSP-1_19_) (red). The nuclei of schizont were stained with DAPI (blue) and slides were visualized by fluorescence microscope. All apical marker proteins and PfMA showed punctate staining in schizonts. PfMA was localized in the micronemes as it signal co-stained with micronemal marker EBA-175. The scale bar indicates 2 µm.

To define the sub-cellular localization of PfMA in the merozoites, co-localization studies were performed by immunofluorescence microscopy using antibodies against different parasite proteins known to localize in different apical organelles or merozoite surface such as EBA-175 (micronemes), PfRH2 (rhoptry), apical sushi protein (ASP; rhoptry) and merozoite surface protein-1(MSP-119) [11,12,13,15]. Co-staining with the surface marker MSP-119 showed that PfMA is located at the apical pole of the intra-schizont merozoites and not on the merozoite surface (Figure 3A). PfMA showed a punctate staining that was distinct from the nuclear staining of DAPI but similar to that observed with EBA-175, PfRH2 and ASP. The PfMA signal was observed to co-localize with that of EBA-175 suggesting that PfMA may reside in the micronemes (Figure 3B). This was consistent with the observation that PfMA staining was very distinct from that of the rhoptry markers, PfRH2 or ASP confirming that PfMA is not localized in the rhoptries (Figure 3C, Figure 3D).

**Figure 4:**
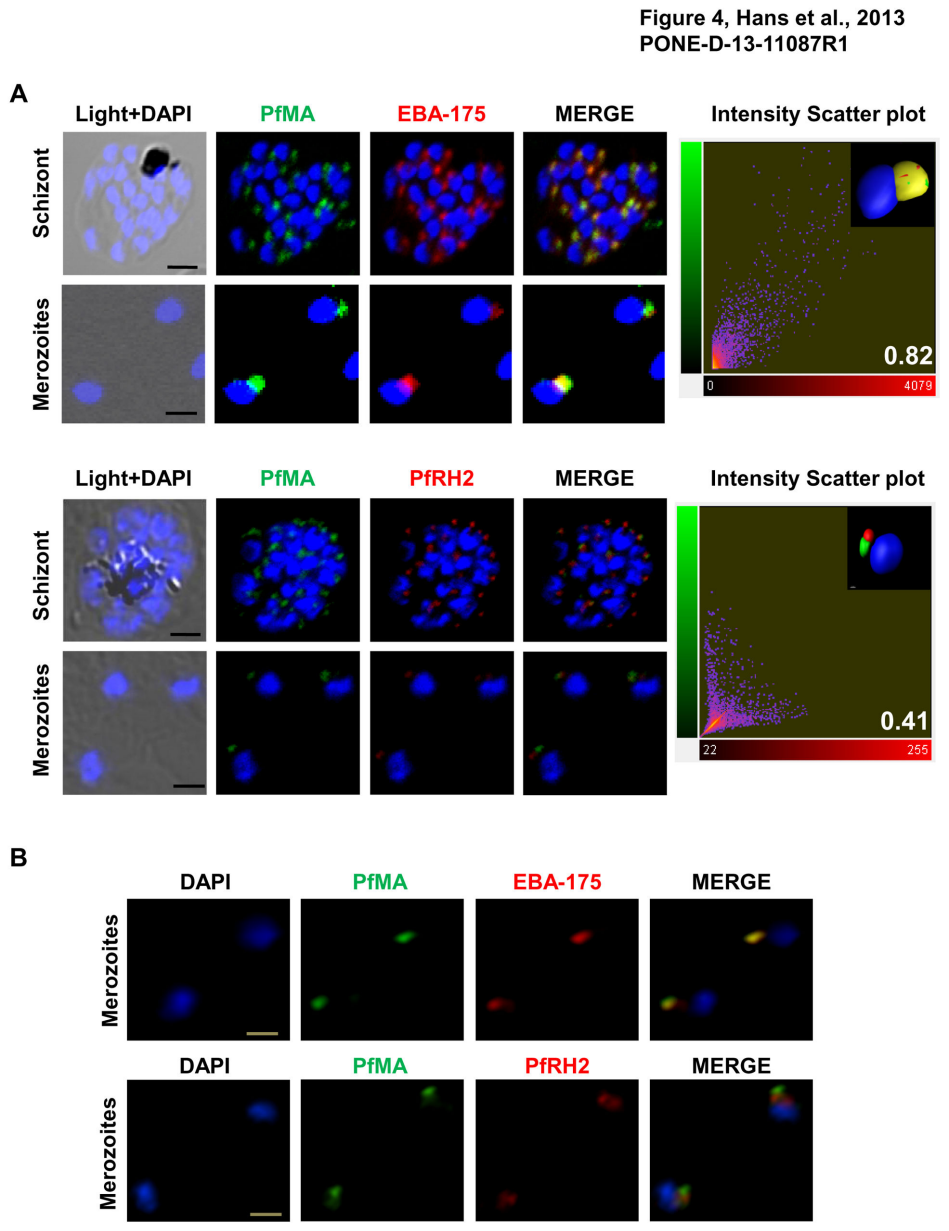
Micronemal localization of PfMA in merozoites analyzed by confocal and super resolution microscopy. (**A**) Co-localization of PfMA was studied with micronemal resident protein EBA-175 and rhoptry protein PfRH2 in mature schizonts and free merozoites by confocal microscopy. PfMA (green) co-localizes with EBA-175 (red) but does not co-localize with PfRH2 (red). [Scale bar 2 µm (schizont); 1µm (merozoite)]. 2D scatter plots of the voxel intensities in the red and green channels as observed for the respective images are shown in the right-hand column. The Pearson’s correlation coefficient was determined for voxel intensity correlation between the red and green channels and is displayed in the lower right-hand corner of the scatter plot. The inset image shows the snapshot of three-dimensional reconstruction of the confocal z-stack images of merozoites (scale bar 0.3 µm). (**B**) 2D Structured illumination microscopy (SIM) also showed complete co-localization between the staining of PfMA and EBA-175 and not with PfRH2, confirming its localization in the micronemes of *P. falciparum* merozoites. [Scale bar 1µm].

Furthermore the co-localization of PfMA was studied by both confocal (A1, Nikon) and structured illumination microscopy (N-SIM, Nikon) with antibodies against the marker proteins, EBA-175 (micronemes) and PfRH2 (rhoptry). The staining of PfMA showed a complete merge with staining of EBA-175 in schizonts and free merozoites (Figure 4A), while it was quite distinct from the staining of PfRH2 (Figure 4A). The z stacks of the confocal images were analyzed by the ‘‘Coloc’’ module of the Imaris x 64 7.2.1 software (Bitplane Scientific). Results of the correlation analysis between the staining intensities observed for the two antibodies were expressed as Pearson correlation coefficient. The analysis revealed coefficients of 0.82 and 0.41 with EBA-175 and PfRH2 respectively (Figure 4A). Smears of free merozoites immunostained with PfMA and EBA-175 and PfRH2 antibodies were also subjected to 2D SIM. SIM provides a resolution in the range of 80-100nm in the XY plane. The staining of PfMA was found to merge with EBA-175 and not with PfRH2, strongly suggesting its localization in the micronemes of *P. falciparum* merozoites (Figure 4B).

**Figure 5:**
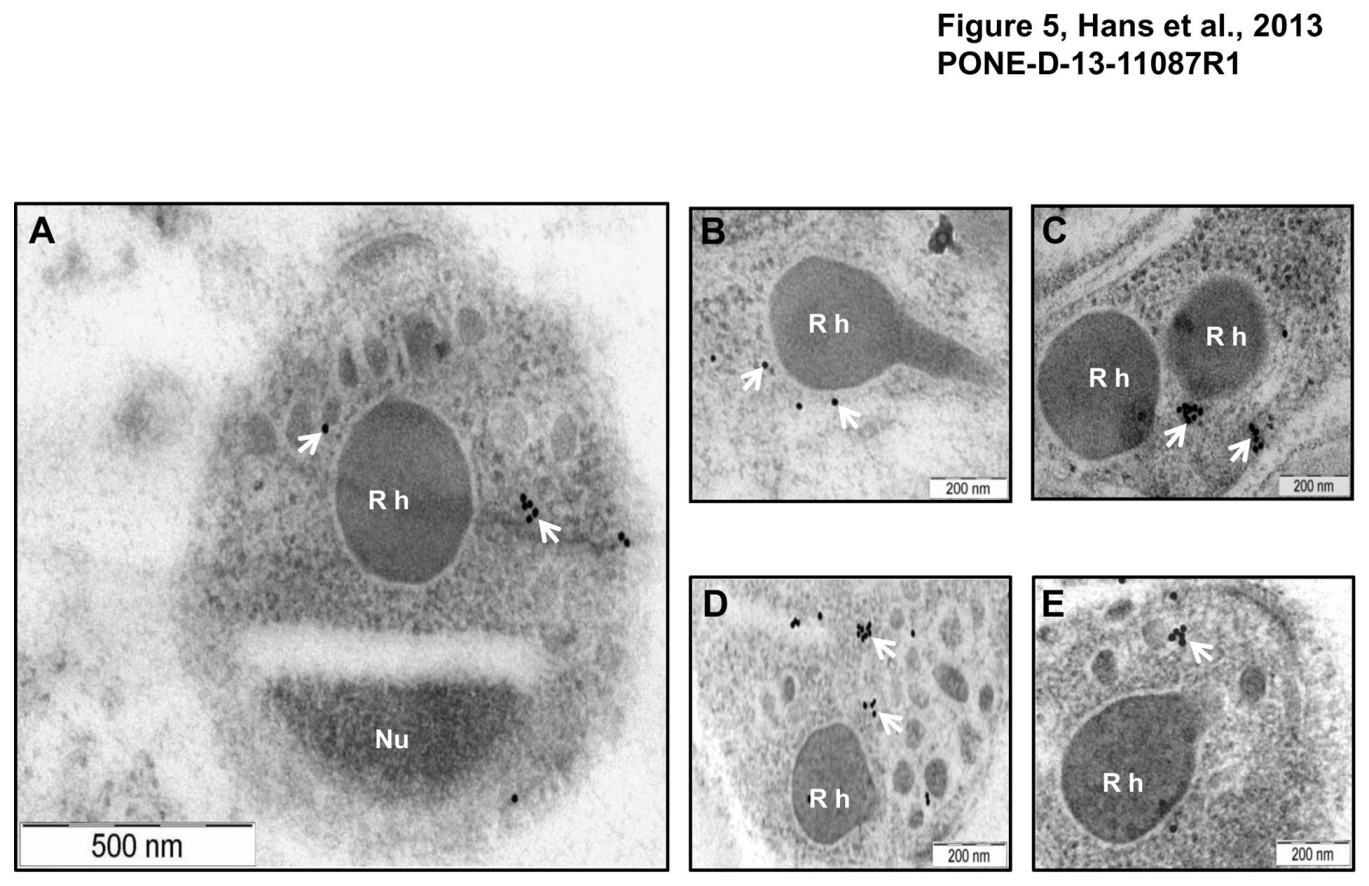
Localization of PfMA by Immuno-electron microscopy. Ultra thin sections of *P. falciparum* parasites at schizont/ merozoite stages were labelled with mouse anti-PfMA or anti-EBA-175 antibodies followed with anti-mouse secondary antibody conjugated with 15nm colloidal gold particle. (**A**) Labelling with the PfMA antibody was confined only to the micronemes (arrows) in the apical end of the free merozoite and was absent from the rhoptry (Rh) or nucleus (Nu). (**B**, **C**) Similarly PfMA staining in the schizonts was observed only in the micronemes and not in the rhoptries. (**D**, **E**) A similar pattern of staining in the schizonts was observed with the micronemal marker EBA-175.

Micronemal localization of PfMA was further confirmed by immuno-electron microscopy (IEM). Ultra thin sections of free merozoites and schizont stage parasites were probed with anti-PfMA mouse antibodies. PfMA staining was specifically detected in small vesicular structures i.e. micronemes at the apical end of merozoites, away from the nucleus and near the rhoptries (Figure 5A). No staining was observed in the rhoptries (Figure 5B; Figure 5C). A similar staining in the small vesicles was also observed with the known micronemal marker, EBA-175 (Figure 5 D, Figure 5E) further confirming that PfMA is located in the micronemes of the *P. falciparum* merozoites.

### PfMA binds to the surface of human erythrocytes

**Figure 6:**
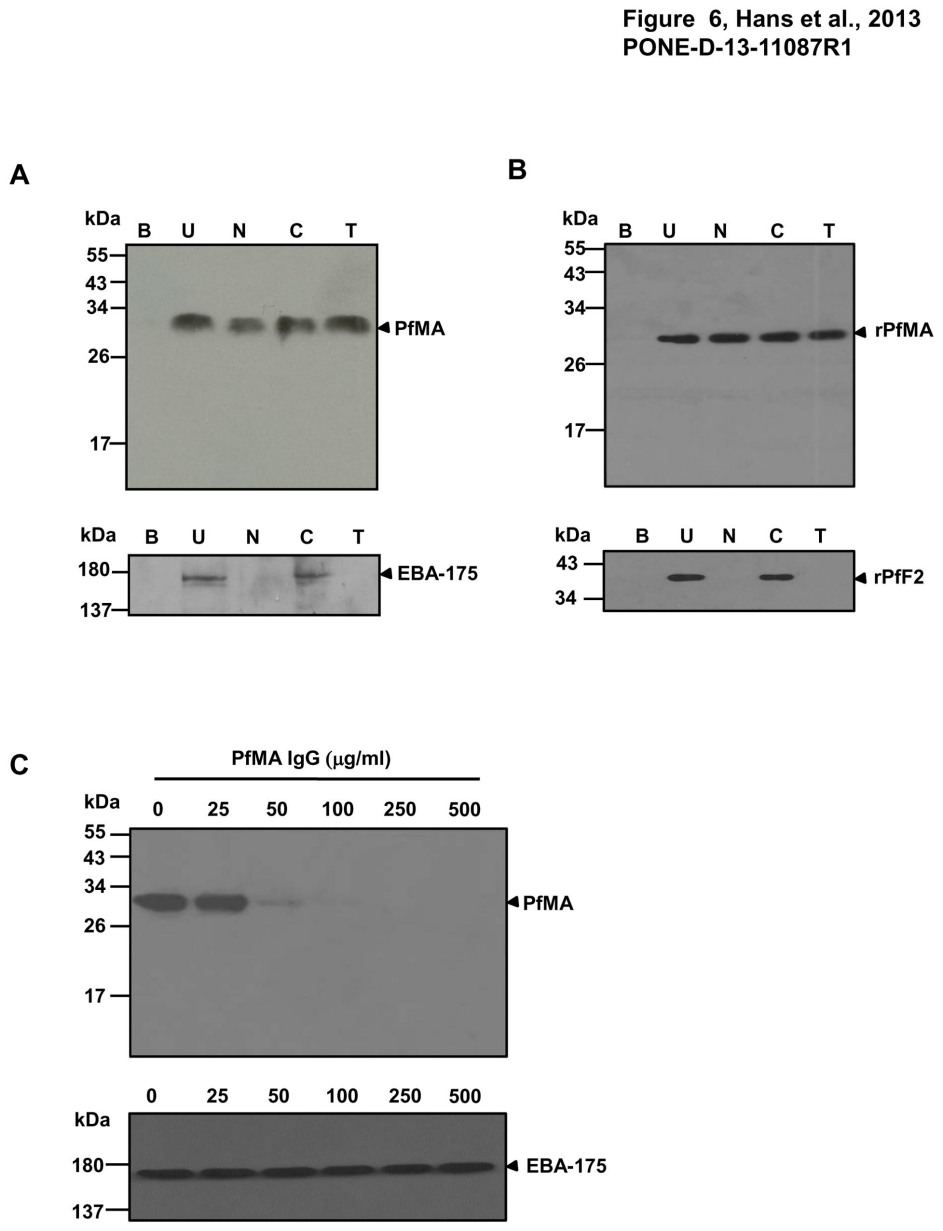
PfMA exhibits erythrocyte binding activity and anti-rPfMA antibodies blocked the erythrocyte binding of native PfMA. (**A**) The erythrocyte binding activity of native PfMA was analyzed with a panel of untreated and different enzymatically treated erythrocytes. The ~32 kDa PfMA protein from parasite culture supernatants bound to untreated (U), neuraminidase (N), chymotrypsin (C) and trypsin (T) treated erythrocytes. Binding of native PfEBA-175 from the same parasite culture supernatants was analyzed as a positive control. (**B**) Recombinant rPfMA exhibited a similar binding specificity with the same set of enzymatically treated erythrocytes. Binding of rPfF2 was analyzed as a positive control. No PfMA protein was detected when the erythrocytes were incubated with an RPMI only control (B). (**C**) Anti-rPfMA antibodies specifically blocked erythrocyte binding of the native PfMA protein. 3D7 culture supernatant was incubated with normal erythrocytes in the presence of purified rabbit anti-PfMA IgG at different concentrations of 0–500 µg/µl. Anti-PfMA IgG had no effect on the binding of the native EBA-175 parasite protein.

The erythrocyte binding activity of the native PfMA protein was studied with untreated and different enzyme treated erythrocytes using culture supernatants obtained from purified *P. falciparum* schizonts in standard assays as reported earlier [12]. The presence of native PfMA among the proteins eluted from the erythrocytes incubated with parasite culture supernatants was detected by immunoblotting using anti-PfMA antibodies. The native ~32 kDa fragment of PfMA was observed to bind to untreated (U), neuraminidase (N), chymotrypsin (C) and trypsin (T) treated erythrocytes (Figure 6A). As a control, RPMI medium containing no parasite protein was incubated with the normal human erythrocytes and no protein was detected in the eluate (B) confirming that the detection of the ~32 kDa PfMA protein was a specific phenomenon. More so, like many invasion proteins that have been reported to be processed during invasion and detected as smaller fragments in culture supernatants [12,14,16,17], we also observed that the PfMA protein in culture supernatants was smaller (3-4 kDa) than that observed in the parasite lysate.

In a similar assay, rPfMA protein was also found to bind erythrocytes with the same specificity as observed for the native PfMA. As a control for the enzymatic treatments, the erythrocyte binding of the native EBA-175 parasite protein from culture supernatants and its recombinant receptor binding domain, PfF2 was also analyzed. As reported earlier, native EBA-175 is known to bind with glycophorin A on the erythrocyte surface and this interaction is sensitive to neuraminidase or trypsin and resistant to chymotrypsin treatment [18,19]. Consistent with this report, both native EBA-175 and recombinant PfF2 bound untreated and chymotrypsin treated erythrocytes but failed to bind with neuraminidase and trypsin treated erythrocytes (Figure 6A, Figure 6B).

### PfMA antibodies block the erythrocyte binding of the native parasite protein

We also determined whether the PfMA antibodies blocked the erythrocyte binding of the native parasite protein. Purified rabbit IgG against PfMA was tested for inhibition of binding in standard erythrocyte binding assay at a concentration range of 25-500µg/ml. The PfMA purified IgG specifically blocked the erythrocyte binding of the PfMA parasite protein with complete inhibition obtained at a concentration of 100µg/ml (Figure 6C). Even at a concentration of 500 µg/ml, PfMA antibodies had no effect on the binding of the native EBA-175 protein further confirming the specificity of the PfMA antibodies (Figure 6C).

### PfMA antibodies exhibit invasion inhibitory activity

**Figure 7:**
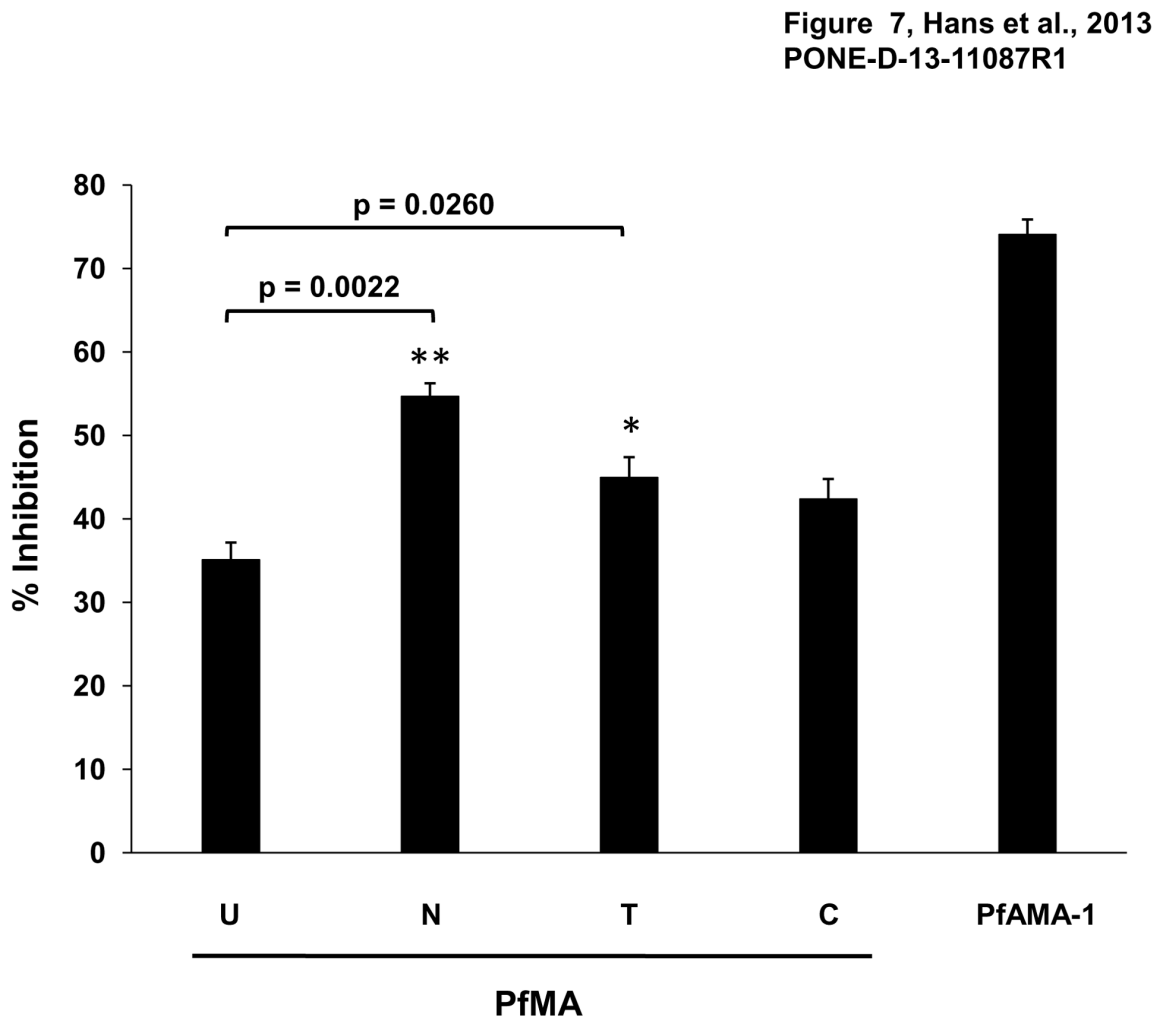
Invasion inhibitory activity of PfMA antibodies. PfMA antibodies were evaluated for their invasion inhibition activity against the *P. falciparum* 3D7 clone. Purified rabbit IgG at a concentration of 20 mg/ml was tested with different enzyme treated erythrocytes. The antibodies exhibited 35% inhibition of untreated erythrocytes (U), 54.6% inhibition of neuraminidase treated erythrocytes (N), 44.95% inhibition of trypsin (T) treated erythrocytes and 42.3% inhibition of chymotrypsin (C) treated erythrocytes. The control PfAMA-1 antibodies at 5mg/ml exhibited 71% inhibition. Three independent experiments were done in duplicate. The error bars represent the standard error of the mean between the assays. Statistical significance between invasion inhibition of untreated and enzyme-treated erythrocytes was calculated by the Student’s t-test with a p value < 0.05 indicating statistical significance (*p < 0.05; ** p < 0.01).

PfMA antibodies were analyzed for their invasion inhibitory activity against the *P. falciparum* clone 3D7 in a one-cycle *in vitro* FACS-based invasion inhibition assay [12]. Purified total IgG against PfMA from rabbit sera exhibited a dose dependent invasion inhibitory activity (Figure S4A) with a 35-37% inhibition at an IgG concentration of 20 mg/ml (Figures S4A and 7). PfAMA-1 antibodies were used as a positive control in the assay and showed 71% invasion inhibition at an IgG concentration of 5 mg/ml (Figure 7). Invasion inhibition of PfMA IgG was also tested with different enzyme treated erythrocytes at 20 mg/ml (Figure 7). With all three enzymatically treated erythrocytes, the invasion inhibitory activity of the PfMA IgG was observed to be higher (54.6% - neuraminidase, 44.9% - trypsin, 42.3% - chymotrypsin) than that of untreated normal erythrocytes (35%) (Figure 7). The experiments were performed thrice in duplicates. The increase in invasion inhibition with the neuraminidase (p=0.0022) or trypsin treated erythrocytes (p=0.026) was statistically significant over the inhibition with untreated erythrocytes as calculated by the student’s t-test (p<0.05).

The invasion of the three enzymatically treated erythrocytes by the 3D7 parasite clone compared to untreated erythrocytes was in the range of 78-80% (Figure S4B), which is consistent with previous reports [20,21]. Thus, the increase of PfMA antibody mediated invasion inhibition with the enzymatically treated erythrocytes was not due to poor invasion of the enzymatically treated erythrocytes by the parasite. The higher invasion inhibitory activity of PfMA antibodies with the enzyme treated erythrocytes is consistent with native PfMA binding with all three enzymatically treated erythrocytes as described above. Thus, restricting the repertoire of receptors accessible to the parasite by enzymatic treatment enhanced the role of PfMA in the invasion of these erythrocytes and as a result increased the inhibitory effect of the PfMA antibodies. Although PfMA antibodies exhibit a low invasion inhibitory activity, the dose dependent increase in invasion inhibition and the higher inhibition observed with enzymatically treated erythrocytes do suggest that the inhibitory effect is specific.

## Discussion

The transcriptome data for *P. falciparum* has provided the much needed impetus to identify novel antigens involved in the crucial process of erythrocyte invasion and validate their potential as vaccine targets [5-7]. With such a large number of genes expressed in the asexual blood stage still remaining uncharacterized, it cannot be denied that the most efficacious blood-stage target antigen for an effective malaria vaccine may have still not been identified. More so, functional characterization of novel parasite proteins many termed as hypothetical remains important for us to get a complete understanding of the biology of the blood stage parasites. On the basis of a bioinformatics approach that involved sorting novel *P. falciparum* genes that may have a putative role in erythrocyte invasion, we have identified and characterized a novel *P. falciparum* protein, PlasmoDB ID: PF3D7_0316000 (www.plasmodb.org) and named it as *P. falciparum* Microneme Associated Antigen (PfMA). A similar approach has been reported previously to identify *P. falciparum* genes involved in the process of erythrocyte invasion [10,22]. PfMA contains an N-terminal hydrophobic sequence that encodes a weak signal, a predicted single transmembrane domain, and a small cytoplasmic tail. PfMA orthologues have been found in different *Plasmodium* species. The structural features of PfMA, it’s timing of expression during the schizont stage and cross species conservation led us to speculate that PfMA could be a potential invasion related protein.

To confirm our hypothesis, we analyzed the expression of PfMA at both the level of RNA transcription and protein translation, and confirmed that its expression was up-regulated during the schizont stage of the parasite life cycle. These findings are in coherence with the transcription data generated from previous microarray studies that placed PfMA in the group of parasite proteins already known or predicted to play a role in the invasion of human erythrocytes [5-7]. Furthermore the expression of PfMA was also detected in 3 other strains of *P. falciparum*.

Our findings on the localization of PfMA by IFA suggested that it is located in the micronemes of merozoites. SIM and immuno–electron microscopy further confirmed that PfMA is resident in the micronemes. Micronemes are vesicles about 160 nm long and 65 nm wide. They are around 40 in number and located at the apex of merozoite [23]. Apart from micronemes, merozoites also possess a secretory network at the apical end comprising of rhoptries and dense granules. These organelles harbour many proteins required for attachment and formation of the junction at the site of parasite entry in the host erythrocyte [2-4,24,25]. Micronemes contain a repertoire of proteins known to be involved in the process of erythrocyte invasion such as the erythrocyte binding antigens (EBA-175, EBA-140,EBA-181), that bind with the host erythrocyte in a sialic acid dependent manner [2-4,11,26-28]. *P. falciparum* microneme proteins are Type 1 integral membrane proteins with a hydrophobic N-terminal signal sequence, a single transmembrane domain and small cytoplasmic tail [29,30,31]. Recently, a GPI-anchored protein GAMA was also found to be localized in the micronemes [14]. PfMA has a single transmembrane domain and a short cytoplasmic tail. However, the prediction of well defined signal sequence is not strong but a stretch of hydrophobic residues at the N-terminus indicates the presence of putative weak signal peptide, which could mediate trafficking through the endoplasmic reticulum-trans golgi network (TGN) [32].

A number of parasite proteins that reside in the apical organelles (Micronemes or Rhoptries) are known to mediate the attachment of the merozoite with the erythrocyte by binding to different erythrocyte surface receptors [2-4,10-12,14,26-31]. Therefore, we evaluated the role of PfMA as an adhesin that binds to human erythrocytes. We demonstrated that PfMA is a parasite adhesin that specifically binds erythrocytes in a sialic acid independent, trypsin and chymotrypsin resistant manner similar to that observed for other parasite ligands such as PfTRAMP, PfRH5 and some variants of BAEBL [16,17,33-35]. The native PfMA protein from culture supernatants observed to bind to erythrocytes was smaller to the one observed in the schizont lysate, which could be attributed to the shedding of PfMA in culture supernatant by cleavage near the transmembrane domain. Similar processing has been observed for several invasion related proteins [12,14,16,17]. In addition, we were able to produce the recombinant PfMA protein that also bound erythrocytes with the same specificity as that of the native parasite protein. Furthermore our results demonstrated that the antibodies against rPfMA specifically blocked the binding of native PfMA to erythrocytes, confirming its role as an adhesin.

We further evaluated the efficacy of antibodies generated against PfMA to inhibit merozoite invasion in a one cycle assay in a dose dependent manner. We tested PfMA purified IgG at a concentration range of 2.5-20 mg/ml as has been reported previously [36,37]. At a total IgG concentration of 20 mg/ml, 35% inhibition of parasite invasion was observed with untreated erythrocytes which further significantly increased with the enzymatic treatments similar to previous reports for other invasion ligands [12,38]. The increase in invasion inhibition efficiency of the PfMA antibodies with enzyme treated erythrocytes is due to the restriction of multiple alternate pathways to the parasite during which PfMA gains a more prominent role as it is still able to bind the enzymatically treated erythrocytes. Thus, the invasion inhibitory activity displayed by the PfMA antibodies is in line with previous reports on other key invasion ligands, whose antibodies have also shown an increase in invasion inhibition with treated erythrocytes compared to untreated erythrocytes [12,38]. The low inhibitory effects of antibodies against parasite ligands is primarily due to the redundancy exhibited by *P. falciparum* due to which it does not remain dependent on any one parasite ligand and possesses the ability to invade erythrocytes through multiple pathways [2-4]. In enzymatically treated erythrocytes, both the erythrocyte receptors and the invasion pathways accessible to the parasite are reduced. Thus, in such a case the parasite proteins whose receptors are resistant to the enzymatic treatments acquire a more prominent role in mediating erythrocyte invasion. This can be further explained by the previously suggested hypothesis of hierarchically placed invasion pathways wherein, the silencing of a dominant invasion pathway results in significant utilization of alternative invasion pathways by the parasite [39-43]. In both scenarios, the antibodies against the key resistant ligand exhibit a higher potency in blocking erythrocyte invasion by the parasite.

The invasion inhibition with the PfMA antibodies is lower compared to that of other previously reported antigens such as PfRH2 [12,44], PfRH5 [45], EBA-175 [46], which could be attributed to the quality of the antibodies raised against the recombinant protein per se or could reflect a secondary role of PfMA among the large number of redundant pathways involved in *P. falciparum* erythrocyte invasion [43]. Our results are similar to those reported with PfRH4, a parasite ligand that binds with CR1 and is known to mediate invasion through the sialic acid independent pathway [38,41,42,47,48]. It was also reported that PfRH4 IgG targeting a 261 amino acid receptor binding region exhibited no invasion inhibitory activity although these antibodies blocked the erythrocyte binding of the native PfRH4 protein [47]. In a later study, PfRH4 antibodies raised against a larger 730 amino acid region also exhibited poor inhibition with untreated erythrocytes but demonstrated potent inhibition with neuraminidase treated erythrocytes [38] as observed in our current study on PfMA.

Thus, following previous studies in which different regions of the same parasite protein have elicited antibodies with variable invasion inhibitory activity, it would be interesting to assess whether a smaller functional region of the PfMA protein would induce more potent invasion inhibitory antibodies. In addition, PfRH4 antibodies have also been shown to exhibit additive inhibition of invasion when targeted in combination with other parasite ligands [49]. Therefore, it would also be important to test whether PfMA antibodies produce any additive or synergistic inhibition in combination with antibodies against other key ligands involved in erythrocyte invasion as reported previously [33,49-52]. These all are interesting lines for future research studies on PfMA, which will further help in validating its potential as a target of antibody mediated blockade of erythrocyte invasion.

In summary, the present study reports a novel *P. falciparum* protein PfMA that is expressed during the schizont stage, resides in the micronemes and binds with the erythrocyte during invasion. Our study adds another novel adhesin to the ever increasing portfolio of *P. falciparum* antigens involved in erythrocyte invasion.

## Materials and Methods

### Ethics Statement

The animal studies described below were approved by the ICGEB Institutional Animal Ethics Committee (IAEC Reference No. MAL52). ICGEB is licensed to conduct animal studies for research purposes under the registration number 18/1999/ CPCSEA (dated 10/1/99).

### Parasite culture

Parasites were cultured *in vitro* as per the standard protocol first reported by Trager and Jensen [53]. The *P. falciparum* strain 3D7 was cultured in O+ human erythrocytes in RPMI-1640 medium (GIBCO) supplemented with 0.5% Albumax II (GIBCO), 24mM HEPES, 360 µM hypoxanthine, 24mM sodium bicarbonate and 10 µg/ml gentamycin at 37°C [53]. Parasite cultures were synchronized by two sorbitol treatments at 48 h intervals as reported previously [54].

### Isolation of DNA and total RNA

The genomic DNA was isolated from *in vitro* culture of *P. falciparum* following a standard protocol [55]. Total RNA was isolated from synchronized *P. falciparum* 3D7 parasite cultures using the mini-RNA isolation kit (Qiagen). 500 ng total RNA was used to synthesize cDNA using the cDNA synthesis kit (Invitrogen) following the manufacturer’s protocol. Gene specific primers were designed for RT-PCR that are as follows: PfMAFwd1 (5’-AACATTCTGAGTAGCCCGTT-3’), PfMARev1 (5’-GGTTCATATTCCTGTTCTTCG-3’), EBA-175Fwd (5’-AATTTCTGTAAAATATTGTG ACCATATG), EBA-175Rev (5’-GATACTGCACAACACAGATTTCTTG-3’), Intron Fwd (5’-GACTTCCACCTTATATTCCATG-3’), IntronRev (5’-TATAAGCCGTAGTTT TATCCCTA-3’), 18S rRNA control primers 18SFwd (5’-CCGCCCGTCGCTCCTACC G-3’), 18SRev (5’- CCTTGTTACGACTTCTCCTTCC-3’).

### Cloning, expression and generation of antisera against PfMA

The region of the native PF3D7_0316000 gene encoding the amino acid sequence of (Met 1 to Lys 244) was PCR amplified from 3D7 genomic DNA using the following primers: PfMAFwd2 (5’-TACGACCCATGGGCATGCACGATTTTT TTTTAAAATC-3’); PfMARev2 (5’-CACGTACTCGAGTCAATGGTGATGGTGATGGT GTTCTAGTTTTTTTAACAAAATATACATGGTT-3’). The PCR product encoding PfMA was digested with NcoI and XhoI (New England Biolabs) and inserted downstream of the T7 promoter in the *E. coli* expression vector pET-28a(+) (Novagen) to obtain the plasmids pPfMA-pET-28a. Sequencing of the ligated plasmids confirmed the correct sequence of the PfMA gene fragments and that the insertions were in the correct reading frame. The plasmids were transformed in *E. coli* BL21 (DE3) cells (Codon plus, Stratagene). Recombinant protein with C-terminus His tag was expressed in *E. coli*, following induction in autoinduction medium (Formedium) for 22 hours. Harvested cell pellets were lysed by sonication and the protein was found in inclusion bodies. The inclusion bodies were collected by centrifugation at 15000xg and solubilized in 6 M Guanidine-HCL. The protein was purified from solubilized inclusion bodies by Ni-NTA (nitrilotriacetic acid) metal affinity chromatography and further diluted 25-fold in an L-Arginine based buffer (50 mM Tris pH 7.4, 10 mM L-Arginine, 2M urea, 150 mM NaCl). The solution was incubated for 24 hours at 4°C with continuous stirring, dialyzed against Tris Buffer Saline (TBS, pH 7.4) and concentrated. The purified protein was processed for in gel trypsin digestion by standard protocols and its identity was confirmed by mass spectrometry.

Group of five Balb/c mice were immunized intra-peritoneally with 25 µg protein emulsified with complete Freund’s adjuvant, CFA (Sigma) on day 0 followed by three boosts emulsified with incomplete Freund’s adjuvant on days 21, 42 and 63. One New Zealand white rabbit was immunized sub-cutaneously with 150µg protein emulsified with CFA on day 0 and boosted intramuscularly twice on day 28 and day 56 with incomplete Freund’s adjuvant. The sera were collected on day 70. Antibody levels were measured in the sera by ELISA.

### ELISA and Immunoblot analysis

Antibody responses in mice and rabbit were quantified by ELISA. Briefly, wells of flat bottom micro titre plates (Nunc) were coated with 200 ng of the recombinant protein in 0.06 M carbonate-bicarbonate buffer, pH 9.6 (Sigma). The plates were washed thrice with 0.05% Tween in PBS (PBST) for 5 minutes each and blocked with 5% skimmed milk in PBS for 1 hour at room temperature. The antigen-coated wells were sequentially incubated with the appropriate dilutions (1:1000-10,24,000) of the respective test sera followed with optimal dilutions of anti-mouse or anti-rabbit horse radish peroxidise (HRP) conjugated secondary antibody (Sigma). The enzyme reaction was developed with o-phenylenediamine as a chromogen and hydrogen peroxide as a substrate prepared in citrate phosphate buffer, pH 5.0 (Sigma). The reaction was stopped with 2N H_2_SO_4_ and the optical density was measured at 490 nm using an ELISA micro-plate reader (Molecular Devices). The experiments were performed twice in triplicate. End point titre was defined as the dilution of the test sera at which OD_490_ was greater than the mean OD_490_ of the pre-immune sera plus two times the standard deviation.

For immunoblot blot analysis, parasites were isolated from tightly synchronized culture by lyzing infected erythrocytes with 0.15% saponin. Parasite pellets were re-suspended in RIPA buffer (100mM Phosphate buffer pH 7.2, 150mM NaCl, 1% NP40, 0.5% sodium deoxycholate, 0.1% SDS, 50mM EDTA and 1mM PMSF) and resultant lysate was quantified by BCA protein estimation method (Pierce). Equal amount of total protein (30µg) from each stage was boiled with SDS buffer and separated on a 12% SDS–PAGE gel. The fractionated proteins were transferred from gel onto the PVDF membrane (Millipore) and blocked in blocking buffer (PBS, 5% milk powder) for 2 hours. The blot was washed twice with PBST (0.10% Tween-20) followed by PBS and incubated for 1 hour with primary antibody (mouse anti-PfMA) diluted in dilution buffer (PBS, and 1% milk powder). Later, the blot was washed and incubated for 1 hour with appropriate secondary antibody (anti-mouse, 1:2500) conjugated to HRP. Bands were visualized by using the Super Signal West Pico ECL detection kit (Pierce).

### Immunofluorescence assay (IFA)

IFAs were performed on the *P. falciparum* 3D7 clone as described earlier [10,13]. Thin smears of schizont, trophozoite, and ring stage parasites were made on glass slides, air dried and fixed with methanol (ice cold) for 45 minutes and blocked overnight at 4°C in 3% (wt/vol) BSA in PBS. After blocking, the slides were washed twice with PBS containing 0.05% Tween-20 (PBST) and PBS. Following washing, slides were incubated with anti-PfMA mouse serum (1:50), anti-EBA-175(1:100), PfRH2 (1:100), PfMSP-1_19_ (1:100) and PfASP (1:100) raised in rabbits at 37°C for 1 hour. Slides were washed and incubated with an Alexa Fluor 488 conjugated anti-mouse IgG at dilution of 1:200 (molecular probes, Invitrogen) and Alexa Fluor 594 conjugated rabbit antibody at a dilution of 1:500 for 1 hour. The slides were washed and mounted in ProLong Gold antifade reagent with DAPI (4',6-diamidino-2-phenylindole) (Invitrogen) and were viewed on a Nikon TE 2000-U fluorescence microscope.

### Confocal Microscopy and Image Analysis

For Confocal and Structured Illumination Microscopy A1 and N-SIM Nikon microscope was used. All images were collected as 3D data sets (z-stacks) with a step size of 0.1 µm between the 21 successive optical sections. De-convolved images were saved and analyzed through Imaris image analysis software (version Imaris x 64 7.2.1, Bitplane Scientific). For the ease of presentation, images in this study are displayed as maximum projection of the 3D image stacks. Co-localization analysis was performed by using ‘‘Coloc’’ module of Imaris which provides functionality for the visualization, segmentation and interpretation of 3D microscopy datasets. The result of co-localization analysis is expressed as a Pearson’s correlation coefficient generated for each co-localization experiment.

### Immunoelectron microscopy (IEM)

For IEM, schizont stage of parasite was fixed for 15 minutes on ice in a mixture of 1% paraformaldehyde-0.1% glutaraldehyde in 0.1 M phosphate buffer (pH 7.4). Fixed specimens were washed, dehydrated, and embedded in LR White resin (Polysciences). Ultra thin sections were blocked at 37°C for 30 minutes containing 2% non-fat milk in water. The grids were then incubated at 4°C overnight with anti-PfMA mouse sera and anti-EBA-175 mouse sera in 1% fish gelatin prepared in 10mM PB (pH 7.2). After washing with 1% fish gelatin, the grids were incubated at 37°C for 2 hours with goat anti-mouse IgG conjugated to 15 nm gold particles diluted 1:50. The grids were stained with uranyl acetate for 2 minutes and examined with a transmission electron microscope (Tecnai).

### Enzymatic Treatment of Erythrocytes

Enzymatic treatments of the erythrocytes were performed by standard protocol described previously [20,46,48]. For Chymotrypsin/Trypsin treatment, 10^9^ erythrocytes in 10 ml of incomplete RPMI (iRPMI) were incubated with TPCK-treated trypsin (Sigma) or TLCK-treated chymotrypsin (Sigma) at a final concentration of 1 mg/ml with rocking at 37°C for 1 hour and then washed with incomplete RPMI (iRPMI). The erythrocytes were then treated with 1 mg/ml of trypsin-chymotrypsin inhibitor (Sigma) at room temperature for 15 minutes. Cells were washed with iRPMI and stored at 4°C for a maximum of 24 hour prior to the assay. For Neuraminidase treatment, erythrocytes (2.5x10^9^) in 5 ml of iRPMI, pH 6.7, were incubated with 0.037 U of *Vibrio cholerae* neuraminidase (Roche) at 37°C for 1 hour, with rocking, and then washed twice with iRPMI.

### Erythrocyte Binding Assays (EBA)

EBAs were performed as described earlier [12,48]. Soluble parasite proteins were obtained from *P. falciparum* 3D7 culture supernatants of schizont-infected erythrocytes as described previously [12,48]. Briefly, culture supernatants were incubated with human erythrocytes at 37°C following which the suspension was centrifuged through dibutyl phthalate (Sigma). The supernatant and oil were removed by aspiration. Bound parasite proteins were eluted from the erythrocytes with 1.5 M NaCl. The eluate fractions were analyzed for the presence of the proteins of interest (PfMA, EBA-175) by immunoblotting using specific antibodies.

### Invasion Inhibition Assay

Invasion inhibition assays with purified rabbit IgGs were performed as described earlier [12,49]. Rabbit IgG were purified using Protein G sepharose (GE Healthcare) and dialyzed against RPMI. In these assays, 6x10^4^ schizont-infected erythrocytes were incubated with 2x10^7^ target erythrocytes along with the purified total IgG in a reaction volume of 100 µl. The efficacy of the antibodies was compared with pre-immune IgG control purified from the pre-bleeds of the same rabbits immunized with PfMA. Parasitemia in cultures was estimated after an incubation of 40 hours (one cycle of invasion in target erythrocytes) by flow cytometry. The whole sample was collected and washed twice with PBS and subjected to staining with ethidium bromide (10 µM) for 15 minutes at room temperature in dark. The cells were washed with PBS, and analyzed by flow cytometry on FACSCalibur (Becton Dickinson) using CellQuest software. Fluorescence signal (FL-2) was detected with the 590 nm band pass filter using an excitation laser of 488 nm collecting 100,000 cells per sample. Following acquisition, data was analyzed for % parasitemia of each sample by determining the proportion of FL-2-positive cells using Cell Quest. The % inhibition was calculated by applying the formula:

% Inhibition = [1-Invasion (PfMA IgG)/Invasion (Pre-immune IgG)]*100

Three independent experiments were done in duplicate. The inhibition data with untreated and enzyme treated erythrocytes was analyzed statistically using Student’s t-test. Statistical analyses were performed using Graph Pad Prism software (version 5.01; Graph Pad Software Inc. USA). Significant difference between two groups was calculated in terms of exact P-values by two-tailed non-parametric Mann–Whitney test. A *p* value < 0.05 was considered statistically significant (Figure 7).

## Supporting Information

Figure S1 **Alignment of PfMA protein sequences from different *Plasmodium* species.** Schematic representation of alignment of PfMA with that of five homologues from P. vivax strain SaI-1 (PVX_095435), *P. berghei* (PBANKA_041380), *P. chabaudi* (PCHAS_041470), *P* *. yoelii* yoelii strain 17XNL (PY03459) and *P* *. cynomolgi* (PCYB_083370). Amino acids that are identical in at least five of six species are coloured in dark, amino acids that are similar in at least three of six species are marked in light grey. The consensus sequence amongst the alignment is also shown.
(TIF)

Figure S2 **Analysis of the purified recombinant rPfMA protein.** (A) Reverse-phase HPLC profile of rPfMA showed that the recombinant protein was purified to homogeneity. (**B**) Mass spectrometric analysis of tryptic digests of rPfMA confirmed it’s identity. *E. coli* expressed and purified PfMA was subjected to in-gel trypsin digestion followed by MALDI TOF/TOF. MS spectra of two identified non-overlapping peptides corresponding to rPfMA are shown.
(TIF)

Figure S3 **Measurement of the antibody responses (end point titers) against rPfMA.** Immunogenicity of rPfMA in (**A**) mouse and (**B**) rabbit was analyzed by ELISA. Sera were serially diluted and assessed for end point titers (blue). Pre-immune sera were taken as controls (red). The data points for the mouse antibodies represent average values of the five mice included in each of the groups. The data points for the rabbit antibodies represent average values of the triplicate readings. Two independent experiments were done in triplicate. The error bars represent the standard error of the mean. (**C**) Immunoblot analysis of rPfMA with immune sera raised in mouse and rabbit (I) in comparison to Pre-immune serum (PI). (**D**) Coomassie stained SDS-PAGE gel, depicting equal loading of lysate samples from the Ring (R), Trophozoite (T) and Schizont (S) stage of the parasite.
(TIF)

Figure S4 **Invasion inhibitory activity of anti-PfMA antibodies.** (**A**) Purified total IgG raised against PfMA in rabbit were tested for their invasion inhibitory activity at a concentration range of 2.5-20 mg/ml. A dose dependent increase in inhibition was observed with 37% inhibition observed at an IgG concentration of 20 mg/ml. Two independent experiments were done in duplicate. The error bars represent the standard error of the mean. (**B**) Invasion of enzymatically treated erythrocytes by the *P. falciparum* clone 3D7 in the absence of PfMA antibodies. In these assays, the control (parasite + treated erythrocytes) was set at initial 0.3% parasitemia and after 40 hours (one cycle) was observed by FACS. The parasitemia was found to be 2.12% for untreated (U), 1.67% for neuraminidase (N) (79% of untreated), 1.70% for trypsin (T) (80% of untreated) and 1.70 in chymotrypsin (C) (80% of untreated) treated erythrocytes. The assay was performed thrice in duplicates. The error bars represent the standard error of the mean.
(TIF)
