# Supplementary figures and images for: Identification and Characterization of a Novel Plasmodium falciparum Adhesin Involved in Erythrocyte Invasion
Source: PLoS One. 2013 Sep 13;8(9):e74790. doi: 10.1371/journal.pone.0074790 (PMC3772933; doi:10.1371/journal.pone.0074790)

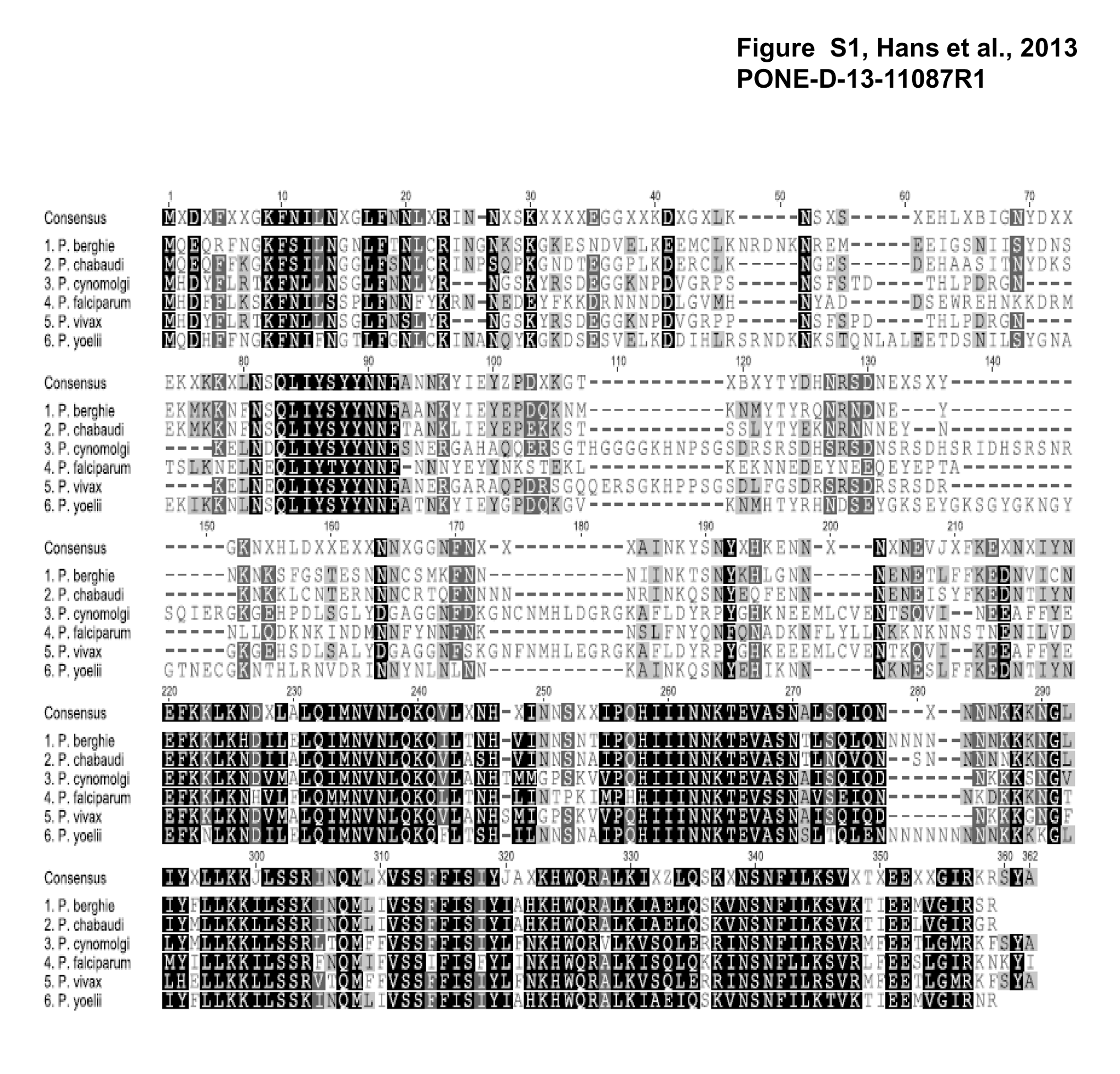

Supplement: Figure S1 — Alignment of PfMA protein sequences from different Plasmodium species. Schematic representation of alignment of PfMA with that of five homologues from P. vivax strain SaI-1 (PVX_095435), P. berghei (PBANKA_041380), P. chabaudi (PCHAS_041470), P . yoelii yoelii strain 17XNL (PY03459) and P . cynomolgi (PCYB_083370). Amino acids that are identical in at least five of six species are coloured in dark, amino acids that are similar in at least three of six species are marked in light grey. The consensus sequence amongst the alignment is also shown. (TIF) [file pone.0074790.s001.tif]

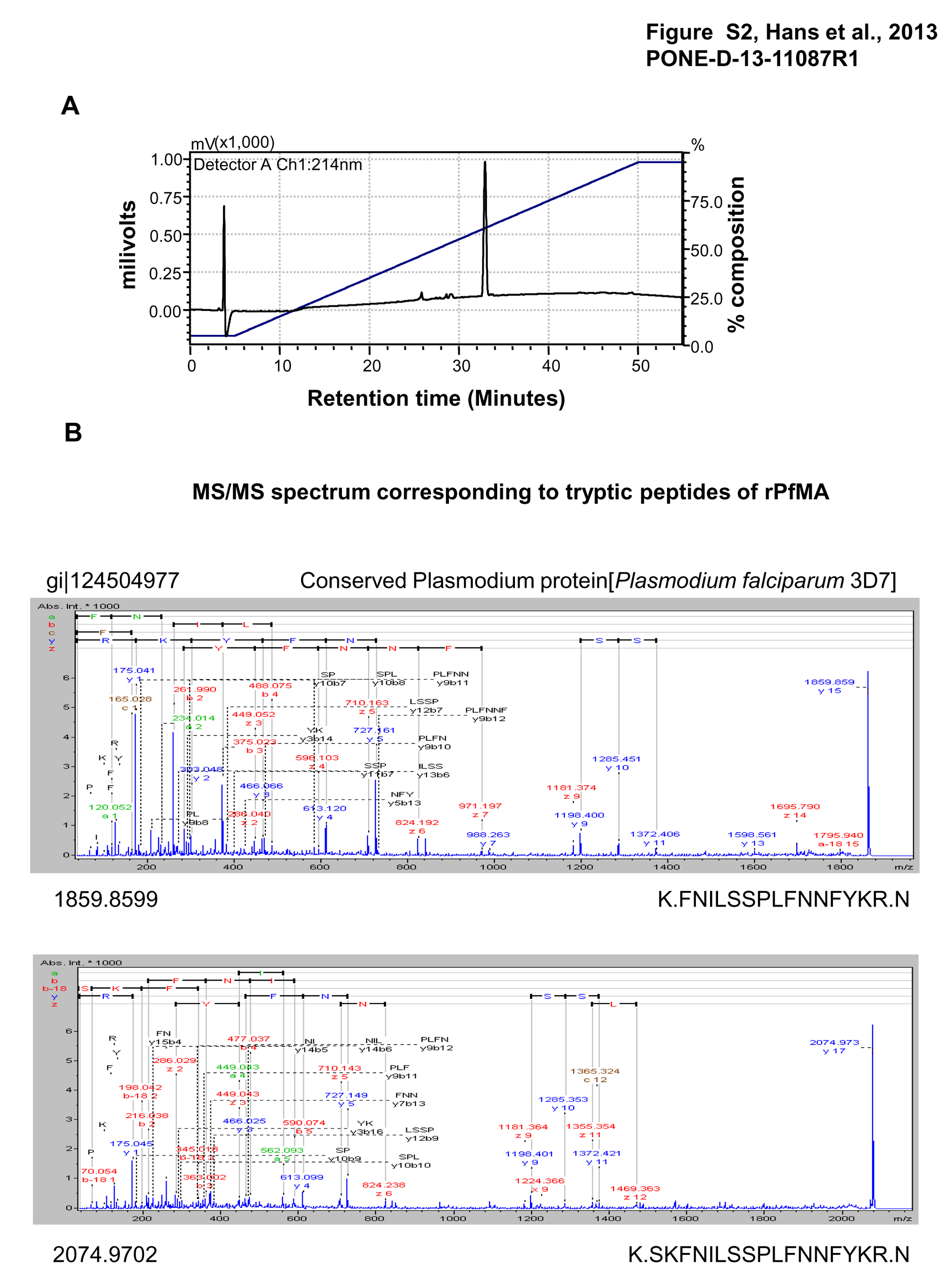

Supplement: Figure S2 — Analysis of the purified recombinant rPfMA protein. (A) Reverse-phase HPLC profile of rPfMA showed that the recombinant protein was purified to homogeneity. (B) Mass spectrometric analysis of tryptic digests of rPfMA confirmed it’s identity. E. coli expressed and purified PfMA was subjected to in-gel trypsin digestion followed by MALDI TOF/TOF. MS spectra of two identified non-overlapping peptides corresponding to rPfMA are shown. (TIF) [file pone.0074790.s002.tif]

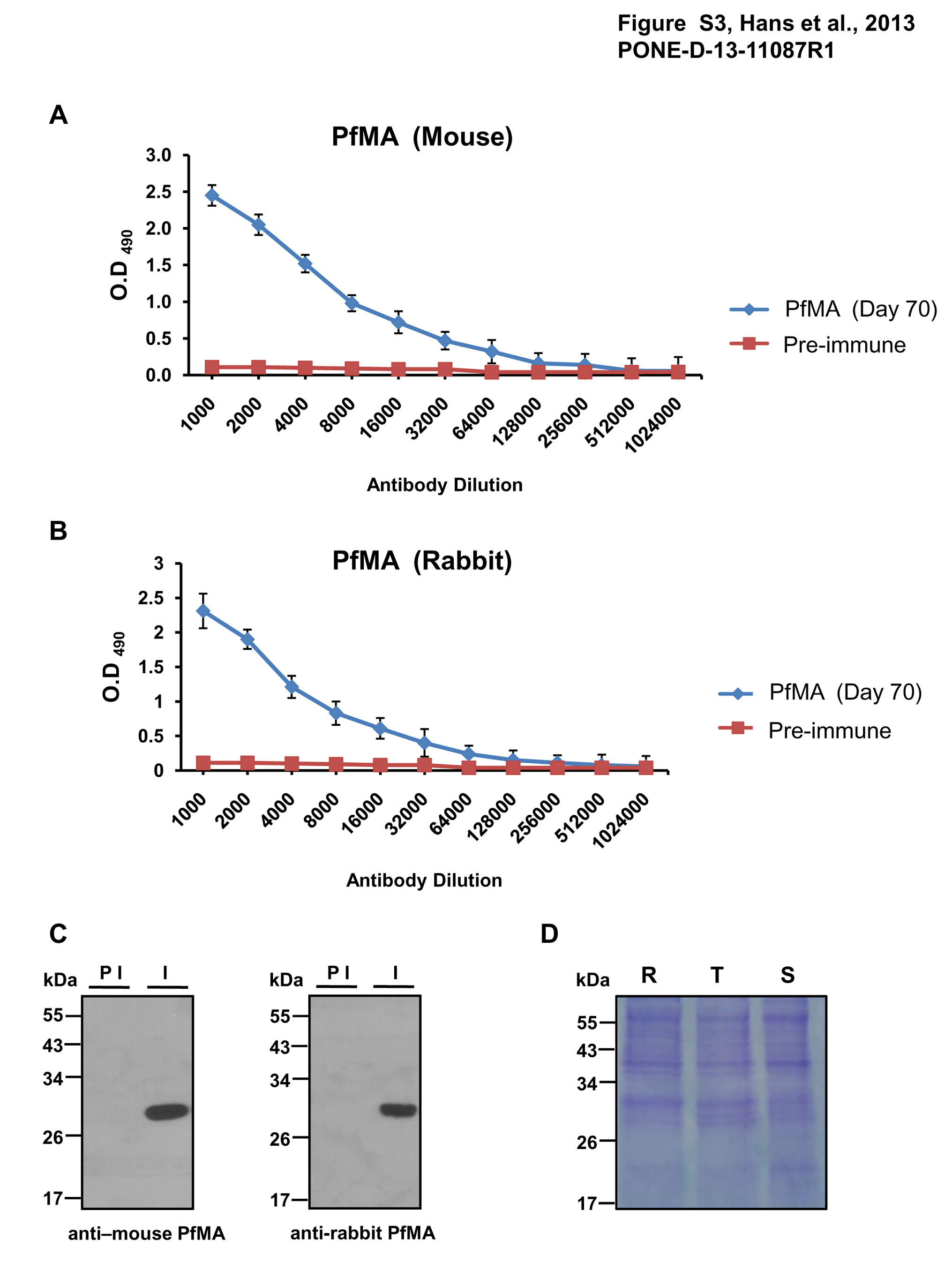

Supplement: Figure S3 — Measurement of the antibody responses (end point titers) against rPfMA. Immunogenicity of rPfMA in (A) mouse and (B) rabbit was analyzed by ELISA. Sera were serially diluted and assessed for end point titers (blue). Pre-immune sera were taken as controls (red). The data points for the mouse antibodies represent average values of the five mice included in each of the groups. The data points for the rabbit antibodies represent average values of the triplicate readings. Two independent experiments were done in triplicate. The error bars represent the standard error of the mean. (C) Immunoblot analysis of rPfMA with immune sera raised in mouse and rabbit (I) in comparison to Pre-immune serum (PI). (D) Coomassie stained SDS-PAGE gel, depicting equal loading of lysate samples from the Ring (R), Trophozoite (T) and Schizont (S) stage of the parasite. (TIF) [file pone.0074790.s003.tif]

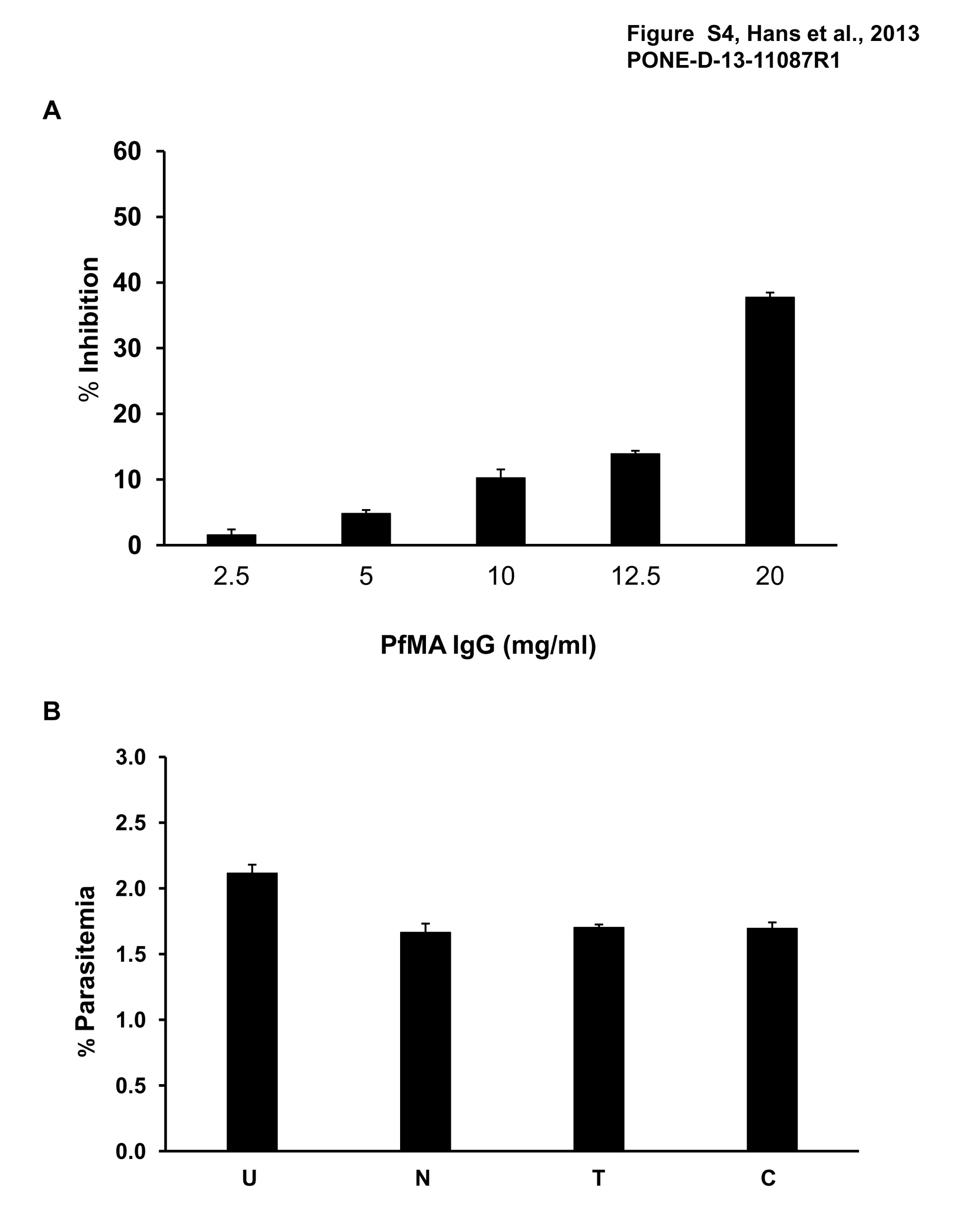

Supplement: Figure S4 — Invasion inhibitory activity of anti-PfMA antibodies. (A) Purified total IgG raised against PfMA in rabbit were tested for their invasion inhibitory activity at a concentration range of 2.5-20 mg/ml. A dose dependent increase in inhibition was observed with 37% inhibition observed at an IgG concentration of 20 mg/ml. Two independent experiments were done in duplicate. The error bars represent the standard error of the mean. (B) Invasion of enzymatically treated erythrocytes by the P. falciparum clone 3D7 in the absence of PfMA antibodies. In these assays, the control (parasite + treated erythrocytes) was set at initial 0.3% parasitemia and after 40 hours (one cycle) was observed by FACS. The parasitemia was found to be 2.12% for untreated (U), 1.67% for neuraminidase (N) (79% of untreated), 1.70% for trypsin (T) (80% of untreated) and 1.70 in chymotrypsin (C) (80% of untreated) treated erythrocytes. The assay was performed thrice in duplicates. The error bars represent the standard error of the mean. (TIF) [file pone.0074790.s004.tif]
